# ‘We need to be supported so that we are able to also provide better care’ Well-being and self-care needs among health workers providing HIV care to children and adolescents in Africa: Qualitative findings from 12 high HIV-prevalence African countries

**DOI:** 10.1371/journal.pone.0335298

**Published:** 2026-05-15

**Authors:** Carley Citron, Lesley Gittings, Megan Oberwasserlechner, Agnes Ronan, Luann Hatane

**Affiliations:** 1 Faculty of Health Sciences, School of Health Studies, Western University, London, Ontario, Canada; 2 Children’s Health Research Institute, London, Ontario, Canada; 3 Centre for Social Science Research, University of Cape Town, Cape Town, South Africa; 4 Paediatric-Adolescent Treatment Africa, Cape Town, South Africa; University of Toronto, CANADA

## Abstract

Frontline healthcare workers providing HIV services to children, adolescents and their families in Africa face significant stressors and well-being related challenges. These in turn may also impact their ability to provide high-quality, compassionate care. Despite healthcare providers’ central role in the delivery of care and support for others, there is limited research exploring their well-being and self-care. This study examined the well-being and self-care-related challenges and needs among healthcare workers providing HIV services to children, adolescents and their caregivers in Africa. A qualitative study design was employed, including participatory priority-setting and focus group discussions with 801 providers across 24 sites in 12 high HIV-prevalence African countries. Data were thematically analysed and Orem’s Theory of Self-Care and the Self-Care Matrix were employed to guide the interpretation and organization of findings. The study identified four major themes. First, participants described burnout and personal struggles as well-being related challenges. Second, they described the belief that addressing these challenges was important for their own well-being, as well as the well-being of their patients. Third, awareness of healthcare worker well-being, alongside effective communication are important contributors to a healthier workforce. Lastly, several interventions were suggested, including fostering teamwork, providing education, and offering psychosocial support within the workplace. Findings emphasize the critical need for tailored interventions that can inform future practices and strategies for healthcare worker well-being. Ultimately, these interventions are crucial for better supporting healthcare providers, enhancing their well-being, and addressing the ongoing challenges in HIV care to children and adolescents in Africa.

## Introduction

Frontline healthcare providers face unique well-being challenges, including workplace exhaustion and high job demands [1]. For these providers to effectively meet patient needs, it has been suggested that they prioritize self-care and maintain their well-being [2]. Because healthcare providers’ work environments impact their mental and emotional well-being, providing targeted support for these needs is critical to sustaining their capacity to deliver compassionate, high-quality care [3].

It has been suggested that healthcare workers providing HIV services to children, adolescent and their families in Africa face well-being challenges due to high-stress environments and the lack of supportive resources in place [4]. In 2020, approximately 1.7 million children under 15 years were living with HIV globally; the majority of whom reside in sub-Saharan Africa [5]. Furthermore, some countries in Southern Africa have rates greater than 100 per 100,000 deaths from HIV/AIDS, and these outcomes are worse for children [6]. A sustained focus on the well-being of frontline providers may enable more resilient health systems in Africa [7] and a more effective, sustainable and holistic response to the paediatric-adolescent HIV epidemic.

Historically, the well-being of frontline health providers has received little attention, though the COVID-19 pandemic brought more awareness to this issue [8]. Global evidence in response to the COVID-19 pandemic presents a further impetus for this work: a 2022 study conducted in Minnesota found that 70% of frontline healthcare workers experienced moderate or moderately severe depression, and 99.2% reported high levels of stress [9]. Furthermore, in Medscape’s 2021 National Physician Burnout and Suicide Report, 42% of physicians reported burnout, and 47% reported a severe impact of their jobs on their personal lives [10]. These numbers indicate a rise in the need for mental health and well-being support and self-care amongst frontline providers. A pronounced effect of COVID-19 was the tremendous impact on the psychosocial well-being of frontline health providers, including those in the paediatric-adolescent HIV response [11]. Furthermore, the pandemic has placed an additional burden and strain on both health systems and healthcare workers themselves [11]. Challenges reported among nurses in South Africa include adequate training, infrastructure, the availability of personal protective equipment, and management support for managing stress and anxiety [12]. Therefore, the pandemic underscored a critical need to prioritise mental well‑being and self‑care within healthcare facilities.

Self-care is a multifaceted concept involving an individual’s ability to maintain mental and physical health and well-being through activities such as exercise, healthy eating, and consistent sleep while avoiding unhealthy behaviors [13]. Reported effective mental health practices at the organizational level include reducing workload and increasing social support, especially from supervisors [14]. A recent publication on the mental health and well-being of frontline providers in the African paediatric-adolescent HIV response documents a high burden of depression among frontline providers, with 39% of participants reporting depression symptomotology, and a relationship between workplace-related quality of life and depression [15]. For healthcare providers in HIV care in particular, taking care of mental and physical well-being are crucial for managing stress, preventing burnout, and ensuring they can deliver effective and compassionate care [16]. However, despite its recognized importance, there remains a significant gap in the literature regarding which self-care interventions have the most impact on healthcare providers’ performance and mental well-being, particularly for those working in paediatric-adolescent HIV care. Furthermore, little is known about the perceptions, experiences, needs and practices of self-care among the paediatric-adolescent HIV providers. This gap highlights the need for further research to identify and evaluate the effectiveness of tailored strategies that can address the unique stressors faced by frontline workers in this field.

Due to being exposed to multiple sources of chronic psychological stress, healthcare workers may constitute a vulnerable population [17]. Specifically, the paediatric-adolescent HIV response in Africa faces significant multi-level challenges, with health providers being overworked, poorly recognized, and inadequately supplied [18]. Further, a lack of time and organizational resources severely impacts the well-being of healthcare workers, in turn affecting the care they can provide [18]. This situation has recently become far more severe due to extensive and far-reaching funding freezes that will disrupt HIV service delivery and reverse gains of the global initiative to end AIDS by 2030 [19]. These recent events have had serious implications for healthcare providers, emphasizing the urgent need to identify feasible, cost-effective, and efficient approaches to support the well-being of frontline providers giving HIV care to children and adolescents.

This paper explores and documents the well-being related experiences, challenges, current practices and needs of frontline providers providing HIV services to children and adolescents in Africa. Implementing effective well-being and self-care practices can strengthen the paediatric-adolescent HIV response by supporting both the well-being of frontline providers and the quality of care delivered to their patients. This paper aims to provide key insights into the contextual realities of well-being practices and their implications for programmatic policy development in healthcare settings.

This study contributes novel insights by centering its focus on the self-care and well-being-related needs and workplace-related strategies of frontline providers; this study is building upon earlier research that presented an overview of frontline provider priorities and strategies to support their empowerment and well-being [20]. By offering deeper analytic engagement with emergent themes, this study extends this prior work and provides a more focused understanding on self-care and well-being of frontline providers that provide care to children and adolescents living with HIV and their families.

## Methodology

This study used qualitative methods with 801 frontline healthcare providers in 24 sites across 12 high HIV-burden African countries during the Paediatric-Adolescent Treatment Africa (PATA) Summit in November 2022 and 2023 [20]. The summit was a gathering of key service providers (i.e., nurses, doctors, peer supporters, community health workers and others such as program staff, NGO workers, researchers, and government officials). Sites included Eswatini (Ezulwini), Kenya (Homa Bay; Nairobi), Malawi (Lilongwe; Blantyre), Mozambique (Maputo; Maxixe City; Inhambane), South Africa (Durban; Johannesburg; East London), Tanzania (Dar es Salaam; Kagera; Tarime), Angola (Luanda), Uganda (Kampala; Soroti), Zambia (Lusaka; Kafue), Cameroon (Yaounde; Route de Mbalayo), Ethiopia (Bahir Dar City), Nigeria (Jalingo; Uyo; Port Harcourt) and Zimbabwe (Harare; Bulawayo). Table 1 below displays the 801 healthcare providers by occupational category.

**Table 1:** Participants, by occupational category.

Participants, by occupational category	
**Occupational category**	**Number of participants**
Nurses	192
Doctors	103
Peer Supporters	192
Community Health Workers	36
Psychosocial Support Workers	109
Other Occupations (i.e., programmers, NGO workers, researchers, government officials)	232
**Total**	**801**

PATA technical leads and frontline providers themselves with facilitation expertise were trained in a series of virtual teams training calls prior to the summit. These members lead facilitation and data collection at the summit. Sites were co-hosted by in-country organisations who can apply to host a summit site and are attended by networks of local clinics, community-based organisations and other key stakeholders.

We employed methods that aimed to understand the contextual realities of frontline providers and to explore what constitutes an enabling and supportive environment to them. This method was designed in order to position participants as experts. Activities were conducted in three sessions over two days in which participants generated, themed, ranked and discussed priorities for what would make them feel empowered and supported in providing frontline paediatric-adolescent HIV services in each of the 24 sites. Participants first engaged in participatory priority-setting in which they generated, themed, and ranked priorities in each group [21]. Group discussions were engaged as a ‘fishbowl focus group’ in which one member of each occupational group was invited to start the discussion to represent their group and share their findings. Other occupational group members were able to replace their group representative to add additional considerations from their perspectives. Themes and priorities were analysed and reported by occupational group elsewhere [22].

Participants provided written informed consent. Ethics approvals were provided by the University of Cape Town’s Centre for Social Science Research (CSSR 2022/04) and Western University (122535). Recruitment for this non-clinical research occurred on the first day of data collection which was the same in each site: 11/21/2022 for one data set and 11/14/2023 for the second data set.

First, participants in each site completed an individual-level word association activity using sticky notes, where they responded in writing to the following prompts: (1) What is difficult about providing paediatric-adolescent HIV and/or SRH services? (2) What would make me feel empowered and supported in providing paediatric-adolescent HIV and/or SRH services? Following this, sticky note responses to these questions were thematically grouped, and a list of themes presented back to participants for verification and discussion. In audio-recorded group discussions, which lasted on average between 50 and 90 minutes, participants discussed these, alongside suggestions for addressing them. Discussions were translated where necessary (the majority were in English, and three conducted in Portuguese, one in French, three in Swahili and one in Amharic). Data were translated and transcribed by transcribers fluent in the language of conversation to English. The English-language dataset was coded. Detailed methods and overall summary findings are reported on elsewhere [22].

Anonymised transcriptions of focus group discussion transcripts were uploaded to Dedoose, a qualitative data analysis software [23], and analysed using a thematic approach, following Braun and Clarke’s (2006) model [24]. Coding was conducted independently by multiple researchers using a predefined coding framework to facilitate thematic mapping. Discrepancies were addressed through iterative team discussions to achieve consensus on code application and theme development. Throughout the process, reflexivity was practiced, and verification procedures were employed to enhance analytic rigor. This was done first by the coding team verifying each other’s work, and second by meetings with the PATA team which is comprised of health workers and experts. Final themes were subsequently refined and validated by the coding team to ensure credibility and consistency. Braun and Clarke’s (2006) [24] thematic analysis was utilized alongside codebook thematic analysis. Codebook thematic analysis is a structured, partly deductive qualitative method that uses a predefined, organized list of themes and codes (the codebook) to analyse data [25].

Well-being and self-care emerged as a theme in group discussions across multiple sites and occupational categories. Given the strength of this theme, as well as a need for improved evidence on the well-being related experiences, challenges, current practices and needs of frontline providers providing HIV services to children and adolescents in Africa, the author team decided to conduct further analysis on this data. The lead author generated codes related to well-being and self-care, while generating a list of initial codes related to the topic. These were then refined, named, and grouped into themes in collaboration with the author team, following a modified approach to Braun and Clarke’s (2006)’s [24] thematic analysis method.

## Findings

Four themes emerged from analysis. The first theme focuses on the mental health challenges faced by frontline providers with burnout and the impact of personal stressors as prominent sub-themes. The second theme focuses on perceived benefits described by frontline health care providers in addressing these challenges, including the perception that addressing these challenges can improve both healthcare worker well-being and patient satisfaction. The third theme centers the importance of understanding and communicating about well-being, emphasizing the need for both awareness and the skills and knowledge to discuss well-being initiatives that could improve workplace conditions. Lastly, strategies and priorities to improve well-being were identified, highlighting three critical sub-themes: teamwork, training, and psychosocial support. These strategies were suggested by providers and could potentially address the issues related to self-care and well-being in healthcare settings. Each theme is presented below.

1. **Frontline provider well-being-related challenges**

Healthcare workers in multiple country contexts consistently described well-being-related challenges. A recurring concern was the inadequate well-being support of healthcare workers, as reflected in the high levels of stress and exhaustion reported. Two key subthemes emerged.

First, a key subtheme that emerged across multiple sites were experiences of feeling burnt out, fatigue, and overwhelmed in the workplace. Heavy workload and high pressure were described as contributing factors to these challenges, alongside staff shortages, highlighting the severe impact of stressful working conditions on providers’ well-being.

*“And specifically, more of the concerns that were raised were that, they are burnt out with workloads. So they are working and working and working.”* (Blantyre, Malawi)

*“because if you’re always working, you maybe fatigued you may have burnout”* (Kafue, Zambia)

*“…we suffer from that burnout because one person has to perform a lot of activities”* (Route de Mbalayo, Cameroon)

Second, in addition to burnout, participants frequently discussed the impact of personal struggles with mental health and stress in the workplace, revealing an additional barrier to well-being that may affect their experience in their work environments. Specifically, they spoke about the difficulty of balancing personal stressors, such as anxiety, family concerns, or emotional strain, with the already high-pressure nature of their jobs. These personal challenges exacerbate the overall stress experienced by healthcare providers, highlighting that their personal emotional well-being is intertwined with professional stressors. They emphasized the importance of understanding how each healthcare worker brings their own personality and personal life stressors into the workplace, highlighting the importance of holistic approaches to understanding and addressing their well-being related needs:

*“we know we are dealing with people who have problems, but we are also humans and we have problems, you leave home having your problems, you have stress, you go interact with people, you are helping someone who also has stress,”* (Dar es Salaam, Tanzania)

*“issues outside our workplace you should also try to engage in, at a personal level because as people we are going different uh things. I might come at work with a stress and then we want your work and then I’m thinking of my problems”* (Durban, South Africa)

*“We have our own problems, we have stresses. Relationship stress, there’s a home, there’s, there’s ART. It’s quite a lot.”* (Ezulwini, Eswatini)

Both subthemes emphasized how frontline workers’ well-being is foundational for the care they provide, and that their well-being is not sufficiently addressed in paediatric-adolescent HIV care settings.

1. **Perceived benefits of self-care and improved well-being**

Another theme that emerged strongly from the data was the importance of initiatives to support and improve the well-being of frontline workers providing HIV care to children and adolescents to address challenges described above. Participants emphasized the profound impact that these can have both on their own well-being and the care they provide to their patients. Two subthemes emerged for this theme: first, enhancing healthcare worker satisfaction and, second, improving patient outcomes. They suggested that fostering a supportive environment for healthcare workers’ well-being can provide improvements for the entire healthcare system. This theme highlights their belief in the dual benefit of increasing the well-being of workers for both the quality of patient care, and their own mental and physical well-being, underscoring how healthcare worker and patient well-being are interconnected.

Participants suggested that promoting and supporting frontline health worker self-care could significantly enhance their well-being, and have a direct and positive impacts on their morale, job satisfaction, and productivity. They further emphasized that when healthcare workers are encouraged and supported in taking care of their health and well-being that they can experience a greater sense of balance, enabling them to manage the high levels of stress inherent in their roles. They also described how feeling supported and valued contributes to their improved sense of well-being, alongside increased motivation and engagement. They suggested that when providers feel refreshed, appreciated, and grounded in their work environments, they are not only more productive but also more resilient, ultimately fostering a healthier and more effective workforce.

*“…but if they have time to rest, you find that by the time they go back for work they’ll be more refreshed.”* (Kafue, Zambia)

*“...looking at the working conditions, we felt that if the staff are working in an environment where they are being appreciated, just simple words of appreciation like well done, good job, it goes a long way to actually boost the morale of the staff…”* (Yaounde, Cameroon)

*“kind of award ceremony, it equally helps to improve their sanity at work and even maybe pushes them to be more productive..”* (Nairobi, Kenya)

Additionally, participants emphasized the critical link between their own well-being, self-care, and the quality of care they deliver, suggesting that investing in their well-being would also improve patient outcomes. Providers widely agreed that prioritizing their own well-being enables them to be more present, compassionate, and effective in patient care. Many highlighted that when they feel physically and emotionally well, they are better equipped to support their patients, ultimately leading to improved health outcomes. This connection is particularly crucial in these HIV care settings, where providers face unique emotional and psychological demands such as burnout, personal stressors, and workplace demands. They described that the challenges of working in such settings require strong support systems, emphasizing that feeling valued and supported in their roles directly translates to their ability to offer better care. By fostering a work environment that prioritizes self-care and mental well-being, healthcare systems can enhance both provider satisfaction and patient health, reinforcing the importance of addressing these issues holistically.

*“Yeah so self-care, self-care start with yourself, if you feel you are safe then now you can offer the best of services to, to all of us.”* (Homa Bay, Kenya)

*“Because we will hear them and we will know how to give them warmth, so it’s with this warmth that they will feel satisfied in the good service because it feels good to our patients.”* (Luanda, Angola)

1. **Understanding and communicating about well-being**

Understanding and communicating in the workplace about well-being emerged as a significant theme in the data. Healthcare workers emphasized the importance of not only understanding their own physical and emotional well-being needs but also being able to articulate these needs in their professional environments. Their descriptions extended beyond simply acknowledging stress and exhaustion, encompassing also the need to actively prioritize well-being and advocate for support. They suggested that effective communication of their well-being need would foster a culture of openness and encourage systemic changes that improve both individual and collective well-being. Participants noted that when their own well-being and well-being-related challenges are openly discussed and normalized, it becomes easier to implement supportive strategies because they are more thoroughly understood by colleagues and employers. Moreover, this theme highlights the importance of integrating well-being discussions formally into workplaces, including into workplace policies and training programs. By equipping healthcare workers with the skills, knowledge and confidence to communicate and understand their well-being-related needs, institutions can create environments where providers feel heard, valued, and empowered to improve and maintain their own well-being. Participants suggested that this was crucial not only for them, but also for the care that they provide to patients too. Two sub-themes were present under this theme. First, they described the importance of better awareness of their own well-being, and second, that it was important to discuss well-being in the workplace. Each is described below.

The understanding of well-being emerged as a crucial factor in participant narratives, as an important component of self-care and capacity for healthcare workers to improve their mental health. Frontline healthcare providers emphasized how self-care and well-being must begin with a clear awareness of personal needs, stressors, and boundaries. They suggested that, without this foundational understanding, the implementation of strategies to boost well-being are likely to be inconsistent or ineffective. Participants highlighted that recognizing when they are mentally, emotionally, and physically depleted is the first step in addressing their well-being. Many noted that healthcare environments often prioritize patient care over provider wellness, making it essential for healthcare providers to identify and advocate for their own needs surrounding well-being. This self-awareness was discussed as a vital skill that enables providers to manage stress, prevent burnout, and sustain their ability to deliver high-quality care.

*“I agree that it is possible to enhance greater health and to also be able to improve on the mental health and well-being of both the staff and the supporters”* (Yaounde, Cameroon)

*“To understand, we are actually in peace and at this moment it is necessary”* (Maputo, Mozambique)

The importance of communication about well-being in the workplace emerged as a sub-theme of how to address mental health and well-being in the workplace. Participants emphasized the importance of discussing mental health and well-being openly, among colleagues and within the broader work environment. Participants stressed that open discussions about well-being should extend beyond personal reflection and be actively integrated into workplace practices. Creating spaces for conversations, such as debriefing sessions and peer support groups for frontline health providers, was seen as a crucial step in promoting mental health awareness. They suggested that these discussions would not only allow healthcare workers to share experiences and coping strategies but also help support self-care, reinforcing the idea that prioritizing one’s well-being is an essential part of delivering quality care. Participants described their belief that by encouraging transparent communication, healthcare workers could advocate for necessary changes, such as manageable workloads, access to mental health resources, and policies that promote work-life balance. They also suggested that it creates an environment where healthcare providers feel comfortable expressing concerns, learning from mistakes, and working collaboratively to improve workplace culture. Ultimately, fostering open dialogue about well-being was suggested as empowering for frontline healthcare providers to support one another, strengthening both individual resilience and the overall well-being of the workplace community.

*“I think also that communication should flow within the healthcare unit… I would like to see the space to vent, the spaces that allow the employees to be able to see what they, they really feel…”* (Maputo, Mozambique)

*“We have listening and supporting each other in time of need. For example, we talk to each other, we can share other problems.”* (Yaounde, Cameroon)

*“Yes, so the debriefing, the meetings within the workplace. Discuss about the challenges and then how are we going to overcome. The thing maybe the mistake, because we are learn by our mistakes guys that a mistake that happened today,”* (Durban, South Africa)

1. **Priorities and strategies for workplace level well-being**

Beyond recognizing and communicating needs for healthcare worker well-being, the implementation of tangible, workplace strategies for psychosocial support and well-being emerged as a key theme. While they suggested that individual-level self-care is essential, participants also highlighted that systemic support from healthcare workplaces is necessary to create a sustainable, healthy work environment to contribute to well-being. They identified several workplace priorities and strategies to improve well-being, including fostering teamwork, providing ongoing training and education, and offering counseling or psychosocial support. These elements were consistently emphasized as critical solutions for mental well-being among healthcare workers. Fostering teamwork was seen as essential for reducing stress and preventing burnout, alongside promoting better well-being. Participants described how strong team dynamics encourage collaboration and shared responsibility, easing the burden on individual providers. Participants also emphasized the importance of regular training on mental health awareness, stress management, and resilience-building to equip healthcare workers with tools for managing workplace challenges. Institutions were also urged to provide accessible mental health resources, including counseling services and professional psychosocial support, to ensure healthcare workers feel supported in high-pressure environments. By prioritizing these strategies, healthcare organizations can address gaps in self-care, promote a culture of well-being, and improve both provider and patient outcomes. Each is described in more detail below.

### Counselling/psychosocial support

A significant subtheme was the pressing need for counseling and psychosocial support for frontline health providers. Given the high-stress nature of their work, they emphasized the importance of accessible, stigma-free counseling services as a means of fostering resilience, preventing burnout, and promoting self-care. Providers noted that structured mental health support, whether through one-on-one counseling with professional psychosocial counselors or guidance from managers trained to address healthcare workers’ psychosocial needs, could significantly enhance their ability to navigate emotionally demanding situations. They expressed that when mental health needs are addressed proactively and professionally, they feel better equipped to manage workplace pressures, sustain motivation, and maintain a high standard of patient care.

*“And I think the debrief sessions with professional counsellors are very key for mental health as we implement the project for both the managers and the cadre”* (Ezulwini, Eswatini)

*“Because I, I know at a facility you have a room for counselling. You could create a space for those things that you’re talking about.”* (Kafue, Zambia)

*“pairing especially the remix so that they support each other, psychosocial support, and added one was assigning case managers to everyone [inaudible] since they are one of the unstable.”* (Nairobi, Kenya)

### Teamwork/positive work environment

A key subtheme identified by frontline providers for improving well-being was the importance of fostering teamwork and creating a positive work environment. Providers suggested that engaging in activities together outside of work, in addition to collaborating effectively on the job, would strengthen relationships and promote a sense of unity. Such efforts were described as vital for building a supportive and cohesive team, which not only improves worker well-being, but it was also discussed how it enhances outcomes for both healthcare workers and the children they care for. Providers discussed fun, off‑site team‑building activities as an effective way to foster a positive work culture and enhance mental and physical well‑being by encouraging teamwork and exercise. Additionally, working collaboratively in groups to care for patients was emphasized as a way to improve morale, reduce stress, and increase job satisfaction, ultimately creating a more productive and emotionally supported workplace.

*“….inspiring one another to just be in a work environment that fosters team, teamwork and also that fosters love as well as unity and togetherness because if these things are missing in a work environment it starts to affect how we deliver our services especially to the adolescents…”* (Lusaka, Zambia)

*“We can even do activities at work, like even egg races - as stupid as it sounds or as silly as it sounds - it helps to bring a team together.”* (Durban, South Africa)

*“On teamwork we mean delivering good services and better services to our people is not a one man’s task so we need to work as a team with other groups - as you have mentioned them - to see that our people get better services.”* (Kampala, Uganda)

### Training/education

Another significant subtheme that emerged was the importance of educating and building the capacity of healthcare workers, with orientation sessions and training workshops highlighted as strategies to improve well-being in the workplace. Participants suggested that equipping healthcare workers with well-being-related knowledge for both themselves and their patients, would empower them to perform their roles more effectively. Education on mental health, in particular, was emphasized as a crucial aspect, ensuring that healthcare workers are not only prepared to care for others but also equipped to prioritize their own well-being.

*“And also capacity building programs such as mental health capacity building programs should be organized for service providers.”* (Yaounde, Cameroon)

*“We can see that in the current situation, the training will help them with how to proceed. To achieve results in the training meetings, you must be able to show the way forward*.” (Tarime, Tanzania)

*“We said that with skills development, obviously capacity building with mmm, training, mentorship mmm improving the scope of our, our care, workers that we send out into the community to enable them to do their work efficiently and effectively.”* (Durban, South Africa)

## Discussion

This study examined the well-being-related challenges, practices, and needs of frontline providers that provide HIV services to children, adolescents and their families in South Africa. Findings contribute to the growing body of literature on well-being and self-care for frontline health providers, suggesting the importance of psychosocial support, education, and collaborative, team-based approaches. In this study, key challenges such as burnout and personal stressors were highlighted, with workplace level well-being practices showing promising potential to address these issues that were discussed amongst healthcare workers. Specifically, effective communication and understanding of healthcare providers’ needs were recognized as crucial for supporting mental health. This finding is supported by global literature such as Majee et al. (2020) [26] which documents the importance of recognizing frontline provider well-being, within service delivery environments.

The first theme identified in this study related to descriptions of frontline provider well-being-related challenges. This finding corroborates with literature emphasizing the job-related stress and burnout faced by frontline providers in the HIV response. For instance, Kim and colleagues (2018) [18] found that 60% of frontline providers working in HIV care in Malawi met the criteria for burnout, attributing this to job-related stressors and heavy workloads. This study reinforces the idea that burnout is a significant issue and highlights how personal challenges, such as working in unsupportive environments, further exacerbate stress. Similarly, a South African study by Mohangi and Pretorius (2017) [27] showed that social workers supporting children living with HIV experienced high stress, emotional burdens, demotivation, and a lack of emotional and practical support, along with organizational barriers to performance. Extreme levels of stress and burnout among those providing HIV care in Africa were also documented by Atukunda and colleagues (2013) [28]. Further, Makina-Zimalirana et al. (2023) [29] highlight factors contributing to burnout, such as inadequate mental health resources, limited leisure time, and heavy workloads, further underscoring the need for integrating self-care practices alongside material interventions. Nkabinde-Thamae & Downing (2024) [30] highlight prevalent issues such as unconscious self-neglect, compassion fatigue, and a lack of managerial support, further revealing gaps in the understanding of self-care needs within healthcare settings. A recent study of 617 frontline providers providing services to children and adolescents living with HIV found that 39% experienced depression symptoms, related to their workplace-related quality of life [15]. Together, these findings elucidate the extent and nature of workplace-related well-being challenges and emphasize the critical need for well-being and mental health supports for frontline health providers that give support to children and adolescents living with HIV in Africa. They emphasize the urgent need to address the persistent issues of burnout and stress among providers.

The second theme identified from this study was of the importance of self-care, within and outside of the workplace. This agrees with existing literature that self-care can improve both patient outcomes and healthcare worker well-being [31]. Parks and Smallwood (2021) [32] found that slightly less than half of rural HIV healthcare providers rated their self‑care as “fair” or “poor,” indicating a need for greater support in developing strategies to safeguard their personal and professional well‑being. However, there is a gap in the literature from the global south linking provider self-care to improved patient outcomes and healthcare worker well-being, and this is an area where further research is needed. The importance of self-care remains under-researched in global contexts and, as highlighted by the study’s findings, is essential.

Finally, priorities and strategies discussed in this paper support, and build on existing global research. Study participants made practical suggestions on how to improve the well-being and of frontline providers in the paedatric-adolescent HIV response, including focusing on psychosocial support, education to raise awareness about well-being and providing practical tools for self-care, formal counselling and fostering teamwork and collaboration. Findings highlight the significance of teamwork, positive work environments, and education and training in promoting self-care, as key factors in improving healthcare workers’ well-being. This aligns with global literature; for example, Majee et al. (2020) [33] explored the impact of self-management (SM) training on the well-being of rural community health workers in South Africa, showing that such training can significantly enhance healthcare worker well-being. Similarly, MohammadiDogahe et al. (2024) [33] demonstrated how educational training based on the Health Belief Model (HBM) improved self-care behaviors. In previous research with frontline providers that provide HIV services to children, adolescents and their families, [22], our team identified teamwork, training and psychosocial support for frontline health providers. This current research provides greater insight and practical recommendations for action, suggesting the importance of incorporating both positive work settings with teamwork and structured training to enhance frontline provider self-care and well-being.

Atukunda et al. (2013) [27] highlighted resource gaps in HIV-care settings and recommended interventions such as education and support. Our study dovetails with, and adds to these suggestions to emphasize not only material resource gaps, but also the importance of understanding and communicating about self-care. By improving understanding and communication about well-being and self-care, areas that have often been overlooked, this approach can enhance the quality of care delivered, while prioritizing the mental and emotional well-being of healthcare providers.

While much of the current literature on strategies to improve healthcare provider well-being emphasizes online technologies [34] and mindfulness-based techniques [35], these approaches were not suggested by participants in this study, who instead suggested education, formal counseling, and fostering teamwork in the workplace. Similarly, mindfulness interventions were not discussed, though Alexander et al. (2015) [35] found that yoga and mindfulness programs helped healthcare workers reduce emotional exhaustion and depersonalization, key components of burnout. Expanding these mindfulness-based interventions to considering other contextually and culturally relevant interventions could address a gap in self-care interventions for frontline HIV workers in Africa. Findings highlight the urgent need for well-being initiatives that incorporate self-care within health facilities to better support providers who are experiencing significant stress and burnout. Addressing these concerns is critical, as effective communication and a deeper understanding of the challenges faced by frontline providers can guide the development of supportive workplace environments that promote self-care and well-being.

At the height of the COVID-19 pandemic, the World Health Organization offered a set of recommendations to ensure mental health support and decent working conditions for health workers [36], including mental health and psychosocial support, monitoring of health provider well-being at the facility level, encouraging help-seeking, and discussing challenges. This was in response to a well-being crisis among frontline providers; For example, Diop et al. (2022) [37] highlight psychosocial concerns for frontline providers, particularly during the COVID-19 pandemic, supporting the need for psychosocial resources during this high-stress time.

This study also addresses a critical gap by focusing specifically on high-stress settings such as the provision of paediatric-adolescent HIV care in Africa. This need has become even more pressing in light of recent funding cuts to the HIV response, as highlighted by the USAID call to action [19]. The abrupt removal of funding that once served as lifelines for HIV care, ARV distribution, maternal and child health services, and mobile outreach to vulnerable populations has left providers under even greater strain [19]. As they navigate these mounting pressures, it is imperative to equip them with sustainable methods to manage stress and maintain their well-being. Suggestions from this study highlight how interventions that integrate teamwork, education, and provide psychosocial, may provide a cost-effective and sustainable support for this crucial workforce. Moving forward, it is essential to transition from theoretical recommendations to tangible, cost-effective, and feasible solutions that can be implemented in real-world settings. By prioritizing provider well-being, institutions can foster a more resilient healthcare workforce capable of sustaining high-quality care for vulnerable populations.

Together, these findings underscore the urgent need for improved workplace strategies that enhance healthcare worker well-being in the paediatric-adolescent HIV response in Africa, and offer valuable practical insights into the self-care and well-being needs and suggestions of frontline health workers in Africa.

### Theoretical contribution

Relevant to the findings from this study is Orem’s Theory of Self-Care, which includes key components of: (1) acting for and doing for others, (2) guiding others, (3) supporting others, (4) fostering an environment that promotes personal development to meet future demands, and (5) teaching others [38]. Each of these elements was reflected in the strategies and priorities identified by frontline providers in the study. *Acting for and doing for others* was closely tied to the emphasis on teamwork, which was highlighted as essential for maintaining mental well-being and delivering quality care. *Guiding others* aligned with the recommendation for access to counseling and peer support to help providers develop sustainable self-care practices. *Supporting others* was echoed in the call for team-building activities that foster shared responsibility and emotional support. *Fostering an environment for personal development* related to capacity-building and training programs aimed at equipping providers with long-term self-care skills. *Teaching others* emerged in suggestions for ongoing mental health education and orientation, encouraging knowledge-sharing among colleagues. Altogether, Orem’s Theory of Self-Care provides a relevant and comprehensive framework through which the self-care for strategies and priorities proposed by healthcare workers in this study can be understood and implemented to strengthen provider well-being in paediatric-adolescent HIV care.

An additional relevant theoretical framework is the Self Care Matrix (SCM). The SCM has four cardinal dimensions that include (1) self-care activities, which were also identified in this study, to include bonding and teamwork exercises as well as mental health breaks during the workday schedule; (2) self-care behaviors, which in this study included personal recognition of stressors and speaking about them in the workplace to address well-being and stay motivated; (3) self-care context: in this study, suggested self-care activities were provided, alongside fostering a contexts of self-care to include a positive work environment and for those who need it; and, (4) self-care environment [13]. Suggestions for creating a self-care environment in this study include workplace policy and culture such as training and orientation as well as psychosocial supports for healthcare providers. The findings of this study align with several of these dimensions, emphasizing team bonding activities, behaviors aimed at improving quality of care, and a supportive context created through psychosocial support and capacity-building efforts.

The interconnected nature of the matrix forms a framework upon which metrics can be developed and applied. SCM reflects real-world conditions while upholding the core aspects of self-care such as maintaining a healthy diet, exercising, practicing good hygiene, and avoiding risky behaviors like smoking and excessive drinking. Though these elements were not specifically highlighted by frontline providers, they remain critical when considering potential interventions that paediatric-adolescent HIV institutions can build upon. The SCM also supports organizational strategies such as teamwork, capacity building, and fostering a positive work environment. Its straightforward structure and accessible language make the model applicable across various healthcare settings and populations. Furthermore, SCM can be used as a lens by which to view, identify, study and evaluate self-care elements in any health and well-being intervention, independent of the disease category or setting [13]. This flexibility makes it an especially useful framework for paediatric-adolescent HIV care settings in Africa, offering a practical and adaptable foundation for building supportive self-care strategies.

### Limitations

The findings of this study must be considered in light of several limitations. Data were collected with frontline providers in the paediatric-adolescent HIV response in Africa, and thus cannot be generalized to other healthcare settings. Additionally, the study relied on thematic analysis and qualitative data, which limited the ability to quantify outcomes or determine whether the proposed interventions would significantly impact the work environment. Future research on implementing strategies and interventions is needed to provide sufficient evidence supporting the claims and feelings that were displayed in the data transcripts. Furthermore, recall and social desirability biases may be present in this data. However, the occupational and geographic diversity of frontline health workers and the large sample size provide some crucial insight and potential next steps to improve environments for frontline health worker well-being and self-care. The recruitment context (PATA Summit) may have introduced selection bias, as participants attending such meetings may be more engaged, supported, or professionally networked than typical frontline providers. This needs to be considered when determining transferability to other global contexts.

### Implications for policy & practice

Building on the findings and prior research, this study proposes several key implications for creating safe, inclusive, and empowering workspaces for frontline providers at the individual, interpersonal, organizational, and societal levels. At the individual level, it is essential for providers to prioritize their mental well-being by engaging in self-care practices, which might include stress management, social connectedness and healthier work-life balance. At the organizational level, wellness routines should be incorporated into workplace policies. This includes fostering teamwork, providing access to counselling services, and offering education and training to enhance mental health literacy. Organizations could implement these strategies through policies that encourage interpersonal support and collaboration. At the societal level, it is critical to acknowledge the specific needs and challenges faced by frontline workers, particularly in high-stress environments such as in the provision of paediatric and adolescent HIV care in Africa. Public health initiatives should promote self-care practices across countries and workplaces, ensuring that well-being is prioritized not only within organizations but also by the broader healthcare community. Promoting well-being and mental health can ultimately transform how healthcare is practiced, leading to better outcomes for both providers and patients.

### Future directions for research

Future studies should focus on implementing and evaluating interventions such as professional counseling, education programs, and teamwork-based activities in paediatric-adolescent HIV care settings in Africa. Research on the practical application of these interventions would provide empirical evidence to support the qualitative findings from this study regarding the importance of self-care for frontline providers. It is especially urgent to explore cost-effective, feasible interventions that can be implemented in resource-constrained environments, particularly in the aftermath of the global pandemic, where healthcare providers face funding cuts and increased demands [19]. Understanding the direct impact of these strategies on the mental well-being of providers is crucial, as supporting their mental health is essential for enabling them to deliver high-quality care. Future research could also expand beyond Africa to examine the broader applicability of these interventions in different regions and healthcare settings, ensuring a more comprehensive understanding of how to foster provider well-being globally.

## Conclusion

This study examines the importance of well-being for paediatric-adolescent HIV healthcare providers in Africa, highlighting the challenges they face, and their pressing well-being needs and ability to engage in self-care. Findings underscore the critical need for targeted interventions that prioritize provider well-being, as stress and burnout continue to impact their ability to deliver quality care. The qualitative data gathered offers valuable, context-specific insights, providing a foundation for future research and the development of evidence-based strategies to support the mental health of those on the front lines of care. These efforts are particularly critical in the current climate, given the heightened demands on frontline healthcare workers during the COVID-19 pandemic and the recent reductions in financial support for global initiatives to end the AIDS epidemic in Africa [8,38]. Continued research and education on strategies to enhance the well-being of frontline healthcare providers are essential, both to support the providers themselves and to ensure the highest quality of care for the patients they serve.

## Supporting information

S1 File Inclusivity in Global Health Questionnaire. (DOCX)

S2 File Related Manuscript. (PDF)

S3 File Table 1. (DOCX)

S4 File Related Manuscript. (DOCX)
