# Supplementary material for: ‘We need to be supported so that we are able to also provide better care’ Well-being and self-care needs among health workers providing HIV care to children and adolescents in Africa: Qualitative findings from 12 high HIV-prevalence African countries
Source: PLoS One. 2026 May 15;21(5):e0335298. doi: 10.1371/journal.pone.0335298 (PMC13178914; doi:10.1371/journal.pone.0335298)
Supplement: S3 File — (DOCX) [file pone.0335298.s003.docx]

Table 1. Participants, by occupational category

| **Participants, by occupational category** | |
| --- | --- |
| **Occupational category** | **Number of participants** |
| Nurses | 192 |
| Doctors | 103 |
| Peer Supporters | 192 |
| Community Health Workers | 36 |
| Psychosocial Support Workers | 109 |
| Other Occupations (i.e., programmers, NGO workers, researchers, government officials) | 232 |
| **Total** | **801** |
